# Blended Online Intervention to Reduce Digital Transformation Stress by Enhancing Employees’ Resources in COVID-19

**DOI:** 10.3389/fpsyg.2022.732301

**Published:** 2022-03-22

**Authors:** Ewa Makowska-Tłomak, Sylwia Bedyńska, Kinga Skorupska, Julia Paluch

**Affiliations:** ^1^Institute of Psychology, SWPS University of Social Sciences and Humanities, Warsaw, Poland; ^2^Polish Japanese Academy of Information Technology, Warsaw, Poland; ^3^Center for Research on Social Relations, Institute of Psychology, SWPS University of Social Sciences and Humanities, Warsaw, Poland

**Keywords:** digital transformation stress, digital transformation, online intervention, self-efficacy, burnout, COVID-19

## Abstract

Generally, the solutions based on information and communication technologies (ICT) provide positive outcomes for both companies and employees. However, the process of digital transformation (DT) can be the cause of digital transformation stress (DTS), when the work demands caused by fast implementation of ICT are elevated and employees’ resources are limited. Based on the Job Demand-Resources (JD-R) Model we claim that DT, rapidly accelerating in the COVID-19 pandemic, can increase the level of DTS and general stress at work. To reduce these negative effects of DTS, we propose the online intervention aimed to strengthen employees’ resources, such as self-efficacy. In this article we evaluate the effectiveness of the blended intervention, based on cognitive behavioral therapy (CBT) and social cognitive therapy, composed of a prototyped online training (e-stressless) and series of interactive online workshops. In a longitudinal study, we examined the change in DTS, perceived stress at work, attitudes toward DT, self-efficacy and burnout in two time points, before and after the intervention. We compared five groups of participants (558 in total), three groups not qualified (*n* = 417), and two groups qualified to intervention (*n* = 141). Our results revealed that the designed blended intervention decreased DTS and one of the dimensions of burnout, namely disengagement. More specifically, the results showed that in the group of active participants of the blended intervention DTS significantly decreased [*M_*T*1_* = 3.23, *M_*T*2_* = 3.00, *t*(432) = 1.96, *p* = 0.051], and in the group of ineligible participants DTS significantly increased [*M*_*T1*_ = 1.76, *M*_*T2*_ = 2.02, *t*(432) = 4.17, *p* < 0.001]. This research paves way for the creation of blended online intervention which could help in addressing employee digital transformation stress before it starts having adverse effects on employee performance and well-being.

## Introduction

Digital transformation (DT) is a continuous process which is changing the economy and the society in fundamental ways (Meske and Junglas, 2020). In organizations, the DT often takes the form of a rapid and ongoing implementation of new information and communication technologies (ICT) solutions. It requires an organizational change (Verina and Titko, 2019) and instilling a culture that supports the change while enabling the company’s overarching strategy (Mergel et al., 2019; Verina and Titko, 2019). Digital transformation also modifies employees’ overall workplace experience: tasks processing, the workload, the sense of control, and social relations within the organization (Dubois et al., 2014; Cortellazzo et al., 2019).

The COVID-19 pandemic leading to national lockdowns forced a transition to new working conditions almost overnight (Dwivedi et al., 2020; Iivari et al., 2020). The digital transformation has accelerated (Iivari et al., 2020; Priyono et al., 2020). Many employees, for the first time, were strongly dependent on ICT solutions (Leonardi, 2020; Park and Inocencio, 2020) and their current workplace was replaced by a remote one, saturated with ICT solutions to the maximum (Shaw et al., 2020). Consequently, the COVID-19 pandemic necessitated the employees’ adaptation to new working conditions and increased job demands. Therefore, DT in these conditions can be a substantial source of stress in the workplace (Day et al., 2012, 2017; Tarafdar et al., 2015; Legner et al., 2017) for some employees (Tims et al., 2012).

Based on the Job Demand-Resources (JD-R) Model (Demerouti and Bakker, 2011), we claim that digital transformation demands (Day et al., 2012) are rapidly growing in the COVID-19 pandemic and they increase the level of digital transformation stress (DTS) (Makowska-Tłomak et al., 2022) and general stress at work (Day et al., 2012; Berg-Beckhoff et al., 2017). In the long term, the elevated level of stress might result in the employees’ burnout (Bedyńska and Żołnierczyk-Zreda, 2015; Berg-Beckhoff et al., 2017). Therefore, to reduce these negative effects of DTS, we propose a psychological intervention aimed to strengthen employees’ resources in order to facilitate healthy coping strategies with digital transformation stress. Due to the limited possibilities of direct contact in the COVID-19 pandemic, we proposed self-help online training supported by online group workshops as a blended intervention to help employees in dealing with digital transformation stress.

The psychological Internet-based interventions have been shown to deliver effective treatment for a variety of mental health problems, such as depression or anxiety (Cieslak et al., 2016; Andersson et al., 2019). Internet-delivered cognitive behavior therapy (CBT) has been used for more than 20 years and hundreds of studies have presented its effectiveness (Andersson et al., 2019). In contrast, interventions conceptualized in the stress and cognitive appraisal model (Lazarus and Folkman, 1984), or job demands-resources (JD-R) model (Bakker and Demerouti, 2007) are still relatively uncommon (Smoktunowicz et al., 2021). Hence, we decided to design an online intervention to address the digital transformation stress in the occupational health and well-being context within the dominating theoretical framework based on the CBT (Bond and Hayes, 2002) and Social Cognitive Therapy (SCT) (Bandura, 1989).

In this study, we tested the effectiveness of the blended intervention approach, composed of online training and online workshops. We predicted that this intervention would reduce perceived stress in the workplace (Lesage et al., 2012; Chirkowska-Smolak, 2016), digital transformation stress (Makowska-Tłomak et al., 2021), and job burnout (Dubois et al., 2014; Berg-Beckhoff et al., 2017). Moreover, our aim was to verify the role of self-efficacy, one of the most important employees’ resources (Aesaert et al., 2017; Lloyd et al., 2017) as a possible mediator of the reduction in stress and digital transformation stress. Following previous studies on the online interventions, we focused here not on a general self-efficacy, but on contextual self-efficacy related to coping with digital transformation stress (Smoktunowicz et al., 2021).

To summarize, the main aim of the study was to verify if the online blended intervention is an effective tool in decreasing stress and digital transformation stress, reducing negative attitudes toward digital transformation and burnout. Firstly, we designed a prototype of the online intervention in form of an online training on the Moodle platform, with different activities strengthening self-efficacy and reducing DTS. Secondly, to evaluate the effectiveness of this intervention, we measured general stress at work, DTS, attitudes toward DT, burnout (Smoktunowicz et al., 2019) and self-efficacy (Gam et al., 2016) in two time points: before and after a blended online intervention. Thirdly, we collected the evaluation about our online training in terms of usability (Kopeć et al., 2018; Makowska-Tłomak et al., 2021), effectiveness and attractiveness.

## Materials and Methods

### Study Design

**FIGURE 1:**
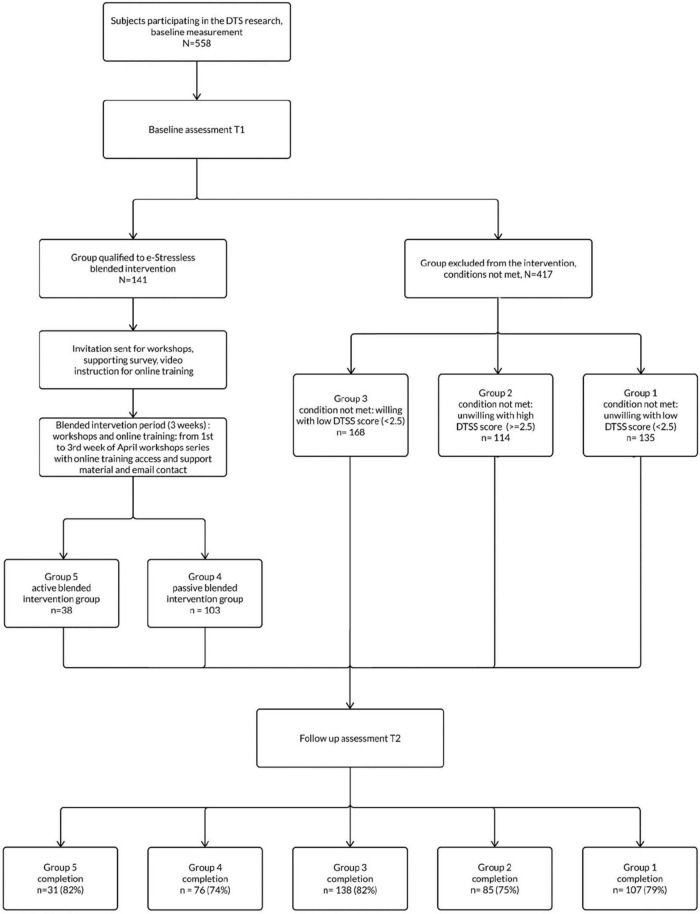
Flow of participants.

The presented study was prepared as a longitudinal study, with two time points, i.e., with baseline assessment (T1), and follow-up assessment (T2)—see flow diagram in Figure 1. The study consisted of two surveys measuring the outcome variables and a blended online intervention, which in turn was composed of online training and workshops (both interactive), as well as support in form of video material. The study was approved by the Ethical Review Board at SWPS University of Social Sciences and Humanities (opinion 8/2021 issued in February 2021).

### Participants

The participants were recruited between March and April 2021, from professionally active adults or students who used ICT technologies at work or studies. The participants represented a large range of occupations: teachers, IT specialists, corporate employees, managers, engineers, from 21 different business sectors (according to the Polish Classification of Business Activities, i.e., PKD). From the convenient sample (*n* = 558) of adults (245 women, 313 men), the following inclusion criteria were applied: (1) Adults, at least 20 years old, (2) using ICT technology at work or studies (3) perceived digital stress level above average (i.e., 2.5 of DTS scale), 4) indicated willingness to participate in workshops and/or a course online (internet intervention). 55% of all respondents (309) declared to participate in the online psychological intervention, but 54% (168) among them qualified to the program because of the higher DTS score. 279 of all survey respondents (50%) represented a higher score of DTS (greater or equal to 2.5) and 60% of them declared their readiness to the intervention program and entered their e-mail.

The invitation to the blended intervention was sent to 141 participants (81 women and 60 men), the average age of 39 (*SD* = 9.8). Although men comprised the majority of the whole study sample, i.e., 56%, this proportion was reversed in the group qualified to the intervention, where women constituted 57% of participants. The demographic characteristics of participants qualified (141) and not qualified to the intervention (417) are presented in Table 1.

**TABLE 1:** Demographic characteristics of the participants qualified and not qualified to the blended intervention.

Variable	Total ineligible participants (*N* = 417)	Respondents qualified to blended intervention (*N* = 141)	Comparison of respondents qualified and not qualified to the blended intervention—tests statistics
Gender N (%)			χ^2^(1, *N* = 558) = 14.05, *p* < 0.001
Females	164 (39.3)	81 (57.4)	
Males	253 (60.7)	60 (42.6)	
Age in years M (SD)	43.43 (10.81)	39.52 (9.88)	*t*(556) = 3.799, *p* < 0.001
Seniority in years M (SD)	19.84 (10.91)	16.09 (9.01)	*t*(556) = 3.680, *p* < 0.001
Remote work N (%)	232 (55.6)	103 (73.0)	χ^2^(1, *N* = 558) = 13.32, *p* < 0.001
Education level N (%)			χ^2^(4, *N* = 558) = 28.64, *p* < 0.001
Primary	3 (0.7)	0 (0)	
Vocational	31 (7.4)	2 (1.4)	
Secondary	170 (40.8)	34 (24.1)	
Studying	6 (1.4)	7 (5.0)	
University degree	207 (49.6)	98 (69.5)	
Self-assessment ICT Skills M (SD)	3.37 (0.91)	3.67 (0.75)	*t*(556) = 3.511, *p* < 0.001
Digital transformation stress—time 1, M (SD)	2.16 (0.76)	3.08 (0.39)	*t*(556) = 13.660, *p* < *0.001*

The blended intervention group consists of 38 participants (active group). Participants who did not decide to take part in workshops and further did not declare the preferred type of intervention have received a notification with educational video material containing information about the online training and access to it. The demographic characteristics of participants are presented in Tables 2, 3.

**TABLE 2:** Demographic characteristics of the participants eligible to the blended intervention.

Variable	Respondents who actively participated in the blended intervention (*N* = 38)	Respondents who received educational materials (*N* = 103)	Means comparison of respondents—blended intervention vs. educational materials—tests statistics
Gender N (%)			χ^2^(1, *N* = 141) = 2.56, *p* = 0.109
Females	26 (68.4)	55 (53.4)	
Males	12 (31.6)	48 (46.6)	
Age in years M (SD)	38.11 (9.80)	40.04 (9.89)	*t*(139) = 1.032, *p* = 0.30
Seniority in years M (SD)	14.53 (8.69)	16.67 (9.09)	*t*(139) = 1.257, *p* = 0.21
Remote work N (%)	30 (78.9)	73 (70.9)	χ^2^(1, *N* = 141) = 0.92, *p* = 0.338
Education level N (%)			χ^2^(3, *N* = 141) = 2.91, *p* = 0.406
Primary	0 (0)	0 (0)	
Vocational	0 (0)	2 (1.9)	
Secondary	6 (15.8)	28 (27.2)	
Studying	2 (5.3)	5 (4.9)	
University degree	30 (78.9)	68 (66.0)	
Self-Assessment ICT Skills M (SD)	3.52 (0.74)	3.72 (0.75)	*t*(139) = 1.48, *p* = 0.14
Digital transformation Stress—time 1, M (SD)	3.18 (0.43)	3.04 (0.36)	*t*(139) = 1.835, *p* = 0.07

**TABLE 3:** Demographic characteristics of the participants ineligible to the blended intervention.

Variable	Wiling but Ineligible participants (*N* = 168)	Reluctant ineligible participants (*N* = 249)	Means comparison of ineligible participants wiling vs. reluctant - tests statistics
Gender N (%)			χ^2^(1, *N* = 417) = 0.65, *p* = 0.422
Females	70 (41.7)	94 (37.8)	
Males	98 (58.3)	155 (62.2)	
Age in years M (SD)	42.13 (10.89)	44.32 (10.68)	*t*(415) = 2.04, *p* < 0.05
Seniority in years M (SD)	18.48 (10.45)	20.77 (11.14)	*t*(415) = 2.11, *p* < 0.05
Remote work N (%)	112 (66.7)	120 (48.2)	χ^2^(1, *N* = 417) = 13.87, *p* < 0.001
Education level N (%)			χ^2^(4, *N* = 417) = 9.18, *p* = 0.057
Primary	0 (0)	3 (1.2)	
Vocational	15 (8.9)	16 (6.4)	
Secondary	56 (33.3)	114 (45.8)	
Studying	3 (1.8)	3 (1.2)	
University degree	94 (56.0)	113 (45.4)	
Self-assessment ICT skills M (SD)	3.69(0.83)	3.16 (0.89)	*t*(415) = 2.04, *p* < 0.05
Digital transformation stress—time 1, M (SD)	1.97 (0.70)	2.30 (0.77)	*t*(415) = –6.17, *p* < 0.001

### Power Calculation

Although the blended intervention composed of online training and online workshops had a limited number of participants, we conducted an *a priori* sample size estimation using G*Power 3.1 3.1 (Faul et al., 2009), to ensure a statistical power of 0.95 to detect the post-test effect of comparisons between study conditions (Smoktunowicz et al., 2021). According to the approach in similar intervention research, we aimed to detect the minimum effect sizes of *d* = 0.30 for the comparisons between conditions at 2 measurement points (T1, T2), while controlling for baseline scores at an alpha error level of 0.05. A power analysis showed that a sample of 38 was needed as minimum. With regard to other online interventions studies (Rogala et al., 2016; Smoktunowicz et al., 2019), we expected a high dropout rate, therefore we decided to qualify a sample of 141 participants, according to baseline conditions. Because of expected high dropout rate as well as approach of prototyping the blended intervention, willingness of participants, and testing in real-life, we decided to use pragmatic trial (Patsopoulos, 2011; Ford and Norrie, 2016; Säfsten et al., 2019; Zvonareva, 2021).

### Procedure

The study flow is presented in Figure 1. The conditions for blended interventions were as follows in the baseline assessment (T1): (1) Willingness, declaration to participate in the blended intervention; (2) Digital Transformation Stress Scale (DTSS) score >= 2.5 (equal or greater mean of DTSS), (3) participants are adult and active professionally, (4) participants have entered their email address. If participants met these conditions, an additional survey was sent where they could choose the type of intervention - blended (workshops with the online course) or only the online course. The participants who have chosen the blended intervention could then choose an available date for online workshops meetings. We sent the invitation to online workshops with proposed slots of online meetings. Before each online workshop, we sent email notifications about the meeting and information about the training online together with the link to our e-stressless online training.

The workshops series (5 online workshops in MS Teams) were conducted from the beginning of April 2021. During each workshop the participants identified the digital transformation stress factors on sticky-cards on Google Jamboard. Participants could add new DTS factors or add to those already mentioned. Afterward, we sent the invitation e-mail with a link to the course online with the key code to the training and the audio-video instruction for logging in (a short movie).

We replicated the approach from the first study (June/August 2020), where we surveyed adult and professionally active people and then selected, from the intervention volunteers, those with high stress indicators (Makowska-Tłomak et al., 2021). After about a month from finishing the blended intervention period, the same group of respondents was tested using the same questions to enable the measurement and comparison of variables. Modification of the questionnaire concerned the removal of questions about the preferred scope of intervention, which were replaced by questions about the participation in the intervention program and preferable module(s) from the training. The list of modules also included those that were not in the online training. We aimed to verify if respondents actually participated in this specific intervention.

We registered the online training users’ activity using standard Moodle functionality (logins, exercises completion, frequency). Additionally, we identified the most active participants during workshops, individual meetings and emails and rated their engagement. We created a supporting variable with the rating of participants’ activity from 0 to 5, where 0 meant *no activity* and 5-*very high activity* at workshops and online training.

All data was collected in online mode only, via a survey. The majority of measured data (T1, T2) was collected by a research agency and, according to prior consent. Simultaneously, data was collected on the Qualtrics platform, under the license of the university. The research application was approved by the Ethical Committee of the University. The present study was conducted in compliance with ethical standards adopted by the American Psychological Association (APA 2010). Accordingly, prior to participation, all participants were informed about the general aim of the research and the anonymity of their data. After marking informed consent to the study, the questionnaire was activated. Participation was voluntary, and participants did not receive compensation for their participation in the study.

### Participatory Workshops

In the study conducted between June and August 2020 we surveyed 150 employees of different sectors to evaluate the level of the digital transformation stress and identify crucial resources protecting from the high level of DT stress (Makowska-Tłomak et al., 2021). Based on the DTS survey results, we distinguished variables that were associated with the DT stress level, i.e., the ICT workload, the ICT hassles. We also identified the self-efficacy, self-assessed ICT competences and ICT Support as significant resources protecting employees from the high level of digital transformation stress. During two series of participatory workshops, we worked with previously selected exercises, which were aimed at strengthening self-efficacy and coping with stressful situations in the workplace during the digital transformation process. The workshops resulted in a list of exercises and materials that were assessed by the participants as most useful for online interventions addressing stress in the workplace (Makowska-Tłomak et al., 2021).

Qualitative assessment of the first series of workshops as well as educational materials and exercise evaluation indicated that co-design workshops can work as psychological interventions themselves. The majority of participants of the first series of workshops admitted that their stress coping knowledge increased and that intervention exercises were useful and helpful to manage DTS and to increase their self-efficacy. During workshops, participants were working with selected exercises, and in the post-workshops survey they indicated the most useful and helpful exercises as well as language and intervention design preferences.

Consequently, we decided to organize the blended intervention as a prototype of an unguided online intervention with educational materials and practical, interactive exercises with social, informative support in the form of interactive workshops. This approach allowed us to collect the feedback of the online intervention prototype focused on dealing with the stress of digital transformation.

### Blended Intervention

Because of the prototype of further unguided online intervention, in the study we opted for the blended online intervention concept, i.e., a mix of social support in form of workshops, consultation meetings and online training (e-stressless), mainly addressing digital transformation stress and perceived stress at the workplace.

E-stressless is a prototype of self-guided online intervention in the form of online training on the Moodle platform (Moodle, 2021). Moodle is a software package designed to help educators create effective online trainings, with a possibility to log users’ activities, self-authorization registration, and privacy policy. The platform is tailored to create exercises in a flexible and effective way. Therefore, we decided to adopt the Moodle platform’s large range of functionalities to the intervention needs.

**FIGURE 2:**
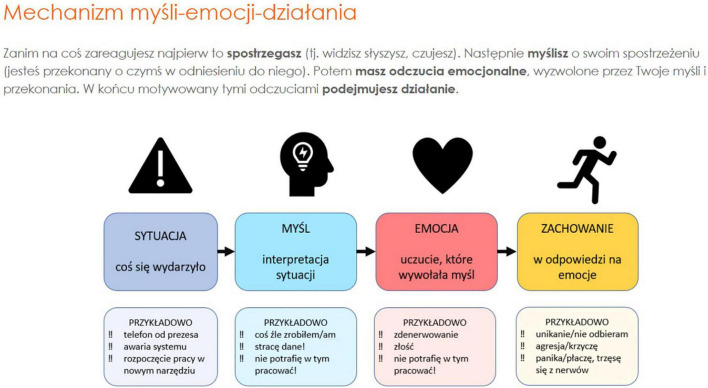
E-stressless online training module 1.

**FIGURE 3:**
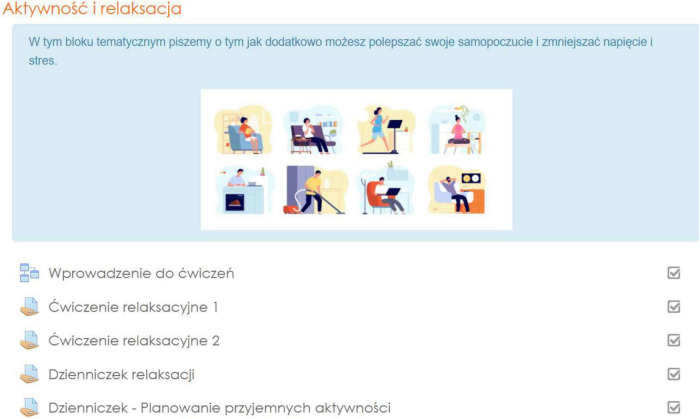
E-stressless online training module 4. Vector image reproduced with permission from vectorstock.com.

The e-stressless online training contains 4 modules with psychoeducational materials and interactive exercises. We adapted the online training intervention to available Moodle functionalities like lessons, quizzes, surveys, essays, with Moodle’s feedback features. These were made available to participants in different variants depending on participants’ needs and preferences. Every module started with a one-page guide for navigation in the module. Each consisted of psychoeducational animated clips and interactive tasks proposing both web-based and offline activities (Smoktunowicz et al., 2021), tips and short TED movies that were made available to participants sequentially (one module a week). We identified two main modules. The first module (1) concentrated on general stress and stress in the workplace. The second module (2) was intended to strengthen the sense of self-efficacy and the ability to cope with difficult situations (see Figure 2). The next two modules were supporting the previous ones—the third module covered relaxation as an efficient method of addressing stress (Figure 3) and the fourth module contained tips and additional materials supporting participants with stress coping. A detailed description of the modules’ content is presented in Table 4. None of the modules were treated as obligatory. All the modules were available for participants for 3 weeks, with full support of the team available. To complete all the tasks within each exercise, participants needed up to 1.5 h. All exercises were available to be retaken depending on individual needs and preferences.

**TABLE 4:** DTS online training—overview of the online intervention on Moodle Platform.

Module	Educational material	Exercises and practical materials
1. Stress in human life	**“**What is stress?”—educational materials as a Moodle lesson, regarding the definitions, causes and consequences of stress, stress at workplace. Materials supported by short TED movies “How stress affects our body and mind.”	- Survey: How much does the stress of digital transformation impact me? - Exercise: “drag and drop”- identification of stressors of digital transformation. - Survey—Does procrastination bother you at work? - Exercise: “Do it Now! How to overcome procrastination.” Exercise with tips and step by step instructions.
2. Overcome difficulties and strengthen yourself	“Different situations: our thoughts, emotions and beliefs”—educational material regarding the thought-emotion-action mechanisms, based on the cognitive–behavioral therapy (CBT). “Self-efficacy belief”—educational material regarding the social cognitive theory (SCT).	- Exercise: “Identifying stressful situations,” quiz form, with instructions to a step-by-step analysis of a chosen situation, with tips. - Exercise: “Get ready for a difficult situation,” quiz form, with instructions to a step-by-step analysis of a chosen situation. - Exercise: “Plan how to deal with difficulties.” - Exercise: “Should I send this?” A list of tips and instructions as a to-do checklist before making a decision. - Exercise: “Goal I want to achieve”—an exercise type to-do task with instructions in form of a checklist.
3. Relaxation and activity	“Exercise’s introduction”—educational material regarding relaxation and activities (like sport, leisure) addressing stress.	- Relaxation exercise 1: “Jacobson training,” progressive muscle relaxation - an audio-visual material with exercise narration. - Relaxation exercise 2: Relaxation according to Benson. - Relaxation diary - an exercise with instruction, describing feelings and emotions during relaxation. - Diary: “Planning leisure activities,” an exercise with instructions, supporting identification and planning of leisure time as a way of coping with stress.
4. Tips and additional materials	“The power of words”—educational material on how the words impact people. Healthy words can improve our mental and physical health. Unhealthy words can be toxic and cause negative thoughts and emotions.	- Exercise: “time management” - an audio-visual material. - Survey: “What factors may cause stress of digital transformation for you”?

The exercises were selected through Cognitive Behavior Therapy (CBT) handbooks such as *Brief cognitive behavior therapy* (Curwen et al., 2018) and *Mind over mood: Change how you feel by changing the way you think* (Greenberger and Padesky, 2015). The selection of exercises was a process started in July 2020 by psychologists before the first series of participatory workshops. Based on workshops participants’ feedback, we selected exercises based on CBT (Beck, 1993) and Cognitive Social Therapy (CST) (Bandura, 1989), empowering self-efficacy and coping with stressful and/or difficult situations. We chose specific exercises for the blended intervention based on the opinions of the participants (from the 2020 workshops and surveys), which have defined the most interesting areas for them regarding coping with stress, especially digital transformation stress.

**FIGURE 4:**
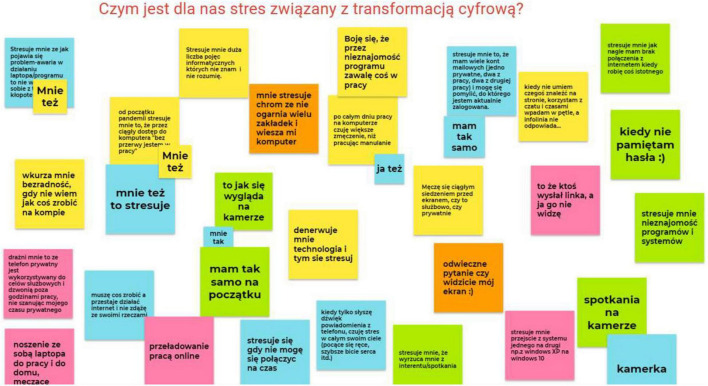
What is digital transformation stress for you—Google Jamboard screenshot.

Before starting with online training, we have organized a series of online workshops which served as a training introduction. Participants were identifying the main digital transformation stress sources and sharing opinions with others using Google Jamboard sticky notes (see Figure 4). Afterward, together with participants we were looking for ways to deal with (digital transformation) stress using a different board. The main aim of these workshops was the introduction to the self-online training, using the digital solution for digital transformation stress. We helped to login to the e-stressless training. We discussed the scope and functionality, strengths and weaknesses of the solution. Participants have been assured that in case of any difficulties, concerns or needs they could always contact us directly, and participate in the next workshops to share their online training opinions.

### Measures

#### Perceived Stress Scale

Perceived Stress Scale (PSS-4), and in the workplace (Cohen et al., 1983; Lesage et al., 2012; Smoktunowicz and Cieślak, 2017). Consisted of four items such as e.g., “How often have you felt difficulties were piling up so high that you could not overcome them?” All items were rated on a 5-point Likert-like scale where 1 meant *Never* and 5 meant *Almost always*.

#### Digital Transformation Attitudes Scale

Digital Transformation Attitudes Scale (DTAS) is a self-descriptive tool for measuring digital transformation stress (Makowska-Tłomak et al., 2021), composed of 12 items. DTAS consists of four subscales concerning three different symptoms of digital transformation stress: (1) Affective (emotional) accompanied by digital transformation in the workplace (3 items, Cronbach’s Alpha = 0.67, e.g., “I am worried that my responsibilities may change and I may not be able to meet them”). (2) Proactive behavior—reactions to the occurring changes in the organization as a result of new ICT solutions implementation (3 items, Cronbach’s Alpha = 0.80, e.g., “I am excited because the changes related to the implementation of new IT solutions will allow me to improve my skills and professional development”). (3) Positive cognitive attitudes, i.e., thoughts and beliefs of ongoing or planned digital, technological or IT changes in the work environment (3 items, Cronbach’s Alpha = 0.88, e.g., “New technologies and ICT solutions are necessary for the efficient functioning of an organization”) 4) Negative cognitive attitudes (3 items, Cronbach’s Alpha = 0.79, an example of the item: “IT implementations of e.g., new systems and programs most often cause chaos in the organization and the growing frustration of its employees”). All items were rated on a 5-point Likert-like scale where 1 = *Not applicable* and 5 = *Applicable* in the first block of statements and 1 = *Disagree* and 5 = *Agree* in the second block of statements.

#### Digital Transformation Stress Scale

Digital Transformation Stress Scale (DTSS) measures the perceived stress of employees during the digital transformation process, in the last month with 6 items (Makowska-Tłomak et al., 2021). An example of item is “How often have you felt irritated in connection to new ICT solutions implementation which have affected your professional duties/tasks?.” All items were rated on a 5-point Likert-like scale where 1 meant Never and 5 meant *Almost always*. Reliability was high with Cronbach’s Alpha = 0.90.

#### Short Occupational Self-Efficacy Scale

Short Occupational Self-Efficacy Scale (Rigotti et al., 2008) was adapted to Polish conditions; it consists of 6 statements measuring self-efficacy related to work with a 5-level response scale ranging from 1 = *Disagree* to 5 = *Agree*. An exemplary item is “I feel prepared for most of the demands in my job.” The reliability of the scale was high with Cronbach’s Alpha = 0.89.

#### Oldenburg Burnout Inventory

Oldenburg Burnout Inventory (OLBI) (Demerouti and Bakker, 2008). The Polish version of OLBI (Baka and Basinska, 2016) measures two dimensions of burnout: exhaustion and disengagement. We used 6 items, 3 from each dimension. Examples of the items are “After work, I tend to need more time than in the past in order to relax and feel better,” and “During my work, I often feel emotionally drained” (both reversed). Participants indicated their answers on a 4-point Likert-like scale where 1 meant *strongly disagree*, and 4 meant *strongly agree*. Reliability of the OLBI was high with Cronbach’s Alpha = 0.79.

#### Self-Assessment Information and Communication Technologies Skills Scale

To assess specific ICT skills, we developed the ICT skills self-assessment scale, based on The Digital Competence Framework for Citizens (Carretero et al., 2017). At the beginning, participants were asked to estimate their general ICT skills in the context of work (“Please evaluate your computer skills in the workplace”), by using 5-point scale where 1 meant *Basic level—limited to elementary functionality* and 5 meant *Very advanced level—programming, graphic processing, computer operation of machine*s. There was also a possibility to mark the answer “*I’m not using a computer at work.*” Afterward, respondents were asked to describe their skills in the listed areas, such as using keyboard shortcuts, or working in different programs commonly used in the workplace. They were also questioned about their activity on the Internet. Examples of items are: “I can prepare a presentation in a dedicated program,” “I can choose the layout, background, template, charts, tables.” “I can pay my bills using online bank transfer.” The responses evaluated their skills on a 5-point scale, where 1 means very low skill level and 5 means very high skill level. The reliability of the Self-assessment ICT scale was high (Cronbach’s alpha = 0.88).

#### Digital Transformation Processing at the Workplace

We asked a question: “Are there any implementation projects (IT) currently being carried out in the organization where you work or study, which affect your work or your activities?”. Respondents indicated their answer by using the following options: *Yes, there are* and *No, there are not, I do not know* and *Not applicable.*

#### Digital Transformation Stress Intervention Expectations

At the end of the survey in the first measurement time (T1: before the intervention) there were 3 questions regarding the scope of intervention and declaration of participation. We asked participants the following question: “Would you like to take part in the online stress counteract program, in particular the digital transformation?”. Participant, who confirmed were asked about their expectation by indicating the areas of interest in the proposal of program for counteracting stress of digital transformation. Respondents who declared to participate in the intervention online, were asked to enter their e-mail address for further contact.

#### Digital Transformation Stress Intervention Usability

At the end of the second measurement time (T2, for all study participants) there was a 2-question block about participation in the blended intervention: “Have you participated in workshops or an online training addressing stress?,” and when the participant has indicated Yes, the next two questions were as follows: (1) “Was the online workshop or training useful for you in coping with stress?,” with a 5-point Likert reverse scale where 1 meant *Definitely helpful* and 5 meant *Definitely unhelpful*; (2) “Which module of online training did you like the most?” with a multiple-choice list with the actual names of online training modules as well as false names of modules.

#### Socio-Demographic Information

Participants were asked to indicate the appropriate year of birth, seniority in years, gender, education level, occupation, and position in their current job.

#### Activity Measure

Activity tracking by Moodle logs reports and an online training list from Moodle online training, intervention survey, Teams list of participation were gathered to evidence blended intervention participants’ activity. Based on these indicators, participants’ activity in the program was evaluated using a 6-point scale where 0 meant *Not applicable* (for DTS study participants who were not selected to the blended intervention program), 1 meant *Lack of activity*, 2—*low activity, 3—moderate activity* (participation in the workshop or/and online training), 4—*high activity* (active participation in the workshop or/and online training) and 5—*very high activity* (many logs in the online training and active participation in the workshops).

## Results

The main goal of the present study was to verify the effect of the psychological intervention aimed at reducing digital transformation stress. Thus, we conducted a series of statistical analyses in which we tested change in several outcome variables: digital transformation stress, digital transformation attitudes, and more general work outcomes such as stress in the workplace, burnout, employees’ resources (i.e., self-efficacy at the workplace). All these variables were measured at two specific time points: before and after the intervention. We applied a two-way analysis of variance in mixed design with between-person factor differentiated 5 groups of participants: (1) not assigned to an intervention, unwilling, with a low DTSS score, (2) not assigned, unwilling, with a high digital transformation stress score, (3) (wait list) not assigned, willing, with a low digital transformation stress score and (4) assigned, willing (with a high digital transformation stress score), not active and (5) assigned, willing (with a high digital transformation stress score), active.

We also conducted a dropout analysis using a chi-square statistic, Mann-Whitney’s *U*-test, and Student’s *t*-test for independent samples. To compare those respondents who participated in the intervention with those who resigned, we tested differences in sociodemographic variables (gender, age, seniority, education level, intervention group) and dependent variables (self-efficacy, digital transformation stress and attitudes, self-assessment ICT skills) measured before the intervention (Time 1). We start the presentation of the results from dropout analysis, and then we present descriptive statistics for all dependent variables and a series of mixed design analysis of variance examining the change in the dependent variables in two measurement points across intervention groups.

### Dropout Analysis

Comparison of groups of respondents revealed significant differences only in age, seniority, education, self-efficacy at work, and one dimension of digital transformation attitude—positive cognition. Those who resigned from participation in the study were younger (dropout *M* = 38.45, *SD* = 9.68, no-dropout *M* = 43.55, *SD* = 10.73), with lower seniority (dropout *M* = 16.71, *SD* = 10.80, no-dropout *M* = 19.50, *SD* = 10.45), lower education level (dropout Mrank = 250.28, no dropout Mrank = 287.59), lower self-efficacy at work (dropout *M* = 3.67, *SD* = 0.72, no-dropout *M* = 3.81, *SD* = 0.66), and higher positive cognition (dropout *M* = 2.23, *SD* = 0.86, no-dropout *M* = 2.05 *SD* = 0.81). Detailed statistics are presented in Table 5.

**TABLE 5:** Statistics of tests in dropout analysis.

Variable	Test statistics comparing dropout and no-dropout
Age	*t*(556) = 4.73, *p* = 0.001, Cohen’s *d* = 0.50
Seniority	*t*(556) = 2.58, *p* = 0.010, Cohen’s *d* = 0.26
Gender	χ^2^(1, *N* = 558) = 0.64, *p* = 0.423
Education	*U* = 22902.5, *p* = 0.011
Intervention group	χ^2^(4, *N* = 558) = 3.95, *p* = 0.413
Self-efficacy at work	*t(*556) = 2.02, *p* = 0.044, Cohen’s *d* = 0.20
DTS	*t*(556) = 0.74, *p* = 0.458
ICT Skills	*t*(556) = 0.51, *p* = 0.609
Stress at work (PSS)	*t*(556) = 0.02; *p* = 0.983
DTAS Affect	*t*(556) = 0.60, *p* = 0.547
DTAS Negative Cognition	*t*(556) = 0.41, *p* = 0.679
DTAS Positive Cognition	*t*(556) = 2.20, *p* = 0.028, Cohen’s *d* = 0.22
DTAS Proactive Behavior	*t*(554) = 2.58, *p* = 0.126

*DTS, Digital Transformation Stress; DTAS, Digital Transformation Attitude Scale; ICT Skills, Self-assessment ICT skills scale.*

The general dropout rate between T1 and T2 equals to 21% (121 respondents). In the 5th group—the active group in the intervention, the dropout rate was 18%—7 participants did not complete the T2 survey, but actively participated in workshops or online training. The highest dropout rate was observed in the 2nd and 4th group—groups with high level of digital transformation stress score before the intervention. The 2nd group was not interested in participating in the blended intervention and the 4th group did not participate actively in interventions and received only video material related to interventions. In the 4th group the dropout rate was equal to 26% (27 participants) and in the 2nd group the dropout was 25% (29 participants). Generally, we can conclude that the dropout level was relatively low compared to others reported in interventions (Rogala et al., 2016; Smoktunowicz et al., 2021).

### Descriptive Statistics

Descriptive statistics: means, standard deviations, and Pearson’s r coefficients for variables in baseline and post-intervention measurement are presented in Table 6. Inspection of the means leads to a conclusion that the level of stress at work, digital transformation stress and burnout is moderate, with values around the middle point of the scale. The level of self-efficacy is rather high. As predicted, self-efficacy is related negatively to stress, digital transformation stress, negative affect, negative cognition toward digital transformation, and both dimensions of burnout. Age and gender were almost non-related to the rest of the variables.

**TABLE 6:** Descriptive statistics: means, standard deviations, and Pearson’s r coefficients for variables in baseline (T1) and post-intervention (T2) measurement.

Variable	M	SD	1.	2.	3.	4.	5.	6.	7.	8.	9.	10.	11.	12.	13.	14.	15.	16.	17.	18.	19.
1. Gender																					
2. Age	42.44	1.708																			
3. DTS -T1	2.39	0.79	−0.15**	–0.04																	
4. DTS -T2	2.45	0.80	–0.07	−0.12*	0.62**																
5. PSS—T1	2.65	0.63	−0.19**	−0.09*	0.45**	0.37**															
6. PSS—T2	2.65	0.66	–0.01	−0.14**	0.35**	0.47**	0.39**														
7. DTAS_B—T1	2.97	0.98	–0.05	–0.00	–0.03	–0.02	0.06	0.07													
8. DTAS_B—T2	2.96	0.97	–0.03	0.07	–0.04	−0.11*	–0.03	0.08	0.54**												
9. DTAS-PC—T1	2.09	0.82	0.00	–0.01	0.21**	0.19**	0.12**	0.20**	0.31**	0.22**											
1. DTAS-PC—T2	2.18	0.85	0.01	–0.01	0.20**	0.20**	0.11*	0.18**	0.22**	0.32**	0.62**										
11. DTAS-AF—T1	2.55	0.83	−0.09*	−0.11*	0.50**	0.42**	0.44**	0.33**	−0.17**	−0.16**	0.11*	0.15**									
12. DTAS-AF—T2	2.55	0.82	−0.10*	−0.11*	0.43**	0.56**	0.28**	0.47**	–0.07	−0.13**	0.17**	0.11*	0.50**								
13. DTAS-NC—T1	3.01	0.85	0.00	0.01	0.40**	0.31**	0.21**	0.17**	0.16**	0.13**	0.13**	0.20**	0.24**	0.26**							
14. DTAS-NC—T2	3.06	0.84	0.01	–0.01	0.35**	0.38**	0.17**	0.26**	0.12*	0.09	0.17**	0.17**	0.20**	0.29**	0.50**						
15. SEW—T1	3.78	0.67	0.04	0.14**	−0.40**	−0.33**	−0.32**	−0.33**	−0.25**	−0.20**	−0.40**	−0.38**	−0.31**	−0.28**	−0.20**	−0.23**					
16. SEW—T2	3.69	0.73	–0.01	0.11*	−0.34**	−0.36**	−0.27**	−0.37**	−0.18**	−0.20**	−0.32**	−0.36**	−0.25**	−0.30**	−0.18**	−0.14**	0.53**				
17. OLBI-E—T1	2.31	0.62	−0.10*	–0.07	0.42**	0.38**	0.39**	0.35**	0.15**	0.12*	0.16**	0.13**	0.32**	0.26**	0.32**	0.29**	−0.31**	−0.25**			
18. OLBI-E—T2	2.39	0.56	–0.08	–0.04	0.36**	0.42**	0.32**	0.39**	0.05	0.08	0.16**	0.17**	0.27**	0.30**	0.30**	0.27**	−0.29**	−0.34**	0.65**		
19. OLBI-D—T1	2.19	0.59	–0.02	−0.20**	0.30**	0.29**	0.31**	0.31**	0.30**	0.24**	0.20**	0.28**	0.27**	0.20**	0.23**	0.23**	−0.46**	−0.36**	0.59**	0.43**	
2. OLBI-D—T2	2.21	0.58	–0.01	−0.15**	0.29**	0.34**	0.23**	0.38**	0.20**	0.21**	0.22**	0.28**	0.25**	0.28**	0.20**	0.19**	−0.33**	−0.45**	0.47**	0.53**	0.66**

**p < 0.01; *p < 0.05.

*PSS, Perceived Stress Scale; DTSS, Digital Transformation Stress Scale; DTAS, Digital Transformation Attitude Scale; DTAS_AF, DTAS Affect; DTAS_PB, Proactive Behavior; DTAS_CN; DTAS Negative Cognition; DTAS_PC, DTAS Positive Cognition; SEW, Self-efficacy; OLBI-E, Burnout—exhaustion, OLBI-D, Burnout –disengagement.*

### Hypothesis Testing

To test the influence of the blended intervention on the level of digital transformation stress and more general work outcomes, we conducted a series of analyses of variance in mixed design with the intervention condition as a between-group factor and pre- and post-intervention measures of the digital transformation stress and work outcomes. Detailed statistics of all effects of analysis of variance are presented in Table 7. Based on theoretical assumptions, we predicted significant interactions of the intervention and time of measurement (pre-post). Therefore, when interaction effect was significant, we present only decomposition of the interaction effect into simple main effects, without further exploration of main effects. Guided by our hypotheses, we also limited description of simple main effects of interaction effect to differences between pre- and post- intervention. The differences between intervention groups in a specific time point and the results of *post hoc* tests for significant main effects of intervention groups are presented in Supplementary Material.

**TABLE 7:** Statistics of the mixed design analysis of variance testing the differences between intervention condition and change in time (pre- and post-intervention).

Variable	Main effect of condition	Main effect of T1-T2	Interaction
DTS	*F*(4, 432) = 91.85, *p* < 0.001, eta^2^ = 0.46	*F*(1, 432) = 0.032, *p* = 0.859, eta^2^ = 0.001	*F*(4, 432) = 12.78, *p* < 0.001, eta^2^ = 0.11
DTAS_PC	*F*(4, 432) = 8.04, *p* < 0.001, eta^2^ = 0.07	*F*(1, 432) = 10.29, *p* < 0.001, eta^2^ = 0.02	*F*(4, 432) = 0.72, *p* = 0.578, eta^2^ = 0.007
DTAS_NC	*F*(4, 432) = 13.55, *p* < 0.001, eta^2^ = 0.11	*F*(1, 432) = 0.39, *p* = 0.535, eta^2^ = 0.001	*F*(4, 432) = 0.91, *p* = 0.460, eta^2^ = 0.008
DTAS_PB	*F*(4, 432) = 13.01, *p* < 0.001, eta^2^ = 0.11	*F*(1, 432) = 0.02, *p* = 0.896, eta^2^ = 0.001	*F*(4, 432) = 0.51, *p* = 0.726, eta^2^ = 0.005
DTAS_NAFF	*F*(4, 432) = 19.25, *p* < 0.001, eta^2^ = 0.15	*F*(1, 432) = 1.16, *p* = 0.281, eta^2^ = 0.003	*F*(4, 432) = 2.16, *p* = 0.073, eta^2^ = 0.020
Stress at work (PSS)	*F*(4, 432) = 14.59, *p* < 0.001, eta^2^ = 0.12	*F*(1, 432) = 0.88, *p* = 0.349, eta^2^ = 0.002	*F*(4, 432) = 1.12, *p* = 0.344, eta^2^ = 0.010
Self-efficacy (SEW)	*F*(4, 432) = 13.77, *p* < 0.001, eta^2^ = 0.11	*F*(1, 432) = 6.14, *p* = 0.014, eta^2^ = 0.014	*F*(4, 432) = 0.98, *p* = 0.420, eta^2^ = 0.009
Burnout—exhaustion (OLBI)	*F*(4, 430) = 10.88, *p* < 0.001, eta^2^ = 0.09	*F*(4, 430) = 3.17, *p* = 0.076, eta^2^ = 0.007	*F*(4, 430) = 1.79, *p* = 0.129, eta^2^ = 0.016
Burnout-disengagement (OLBI)	*F*(4, 430) = 7.39, *p* < 0.001, eta^2^ = 0.06	*F*(1, 430) = 0.33, *p* = 0.568, eta^2^ = 0.001	*F*(4, 430) = 03.75, *p* = 0.005, eta^2^ = 0.034

*Pillai’s trace was reported in all within-group effects. DTS, Digital Transformation Stress; DTAS_PC, Digital Transformation Attitude—Positive Cognition; DTAS_NC, Digital Transformation Attitude –Negative Cognition; DTAS_PB, Digital Transformation Attitude—Proactive Behavior; DTAS_NAFF, Digital Transformation Attitude—Negative Affect.*

The results showed that there were significant interactions of condition and measurement points in DTS, at the tendency level in DTAS—negative affect, and in disengagement—one of the dimensions of burnout. Decomposition of the interaction for DTS showed that there were significant changes in the level of DTS in the following groups: not assigned and not willing to participate in the intervention (1st group), and not assigned but willing with low stress (3rd group). In these groups, the digital stress level was higher in T2 than in T1 [group1: *M*_*T*1_ = 1.76, *SE* = 0.05, *M*_*T*2_ = 2.02, *SE* = 0.07, *t*(432) = 4.17, *p* < 0.001; group 3: *M*_*T*1_ = 1.95, *SE* = 0.04, *M*_*T*2_ = 2.21, *SE* = 0.06, *t*(432) = 4.67, *p* < 0.001]. Participants who were not assigned to the intervention because they were unwilling to do so (with high stress, 2nd group) had a lower stress level in T2 than in T1 [*M*_*T*1_ = 2.70, *SE* = 0.08, *M*_*T*2_ = 2.95, *SE* = 0.06, *t*(432) = 3.44, *p* < 0.001]. As predicted, participants who were actively involved in the intervention (5th group) had a lower level of digital transformation stress in T2 than in T1 [*M_*T*1_* = 3.23, *SE* = 0.09, *M_*T*2_* = 3.00, *SE* = 0.13, *t*(432) = 1.96, *p* = 0.051].

In the DTAS—negative affect, there were significant differences only among participants who were actively involved in the intervention (5th group). They had a lower level of negative emotions related to digital transformation in T2 than in T1 [*M_*T*1_* = 3.20, *SE* = 0.14, *M_*T*2_* = 2.80, *SE* = 0.14, *t*(432) = 2.71, *p* = 0.007]. There were no significant differences in the other groups.

Interestingly, the only change observed in general work outcomes was in one of the dimensions of burnout, namely disengagement. Active participation in the intervention (5th group) lowered the level of disengagement [group 5: *M_*T*1_* = 3.20, *SE* = 0.14, *M_*T*2_* = 2.39, *SE* = 0.11, *t*(430) = 2.59, *p* = 0.010]. Among the participants who wanted to take part in the intervention but were not assigned with low stress (3rd group) the pattern was reversed and their level of disengagement was higher in T2 than in T1 [group3: *M*_T1_ = 2.10, *SE* = 0.05, *M*_T2_ = 2.00, *SE* = 0.05, *t*(430) = 2.61, *p* = 0.009]. There were no significant differences in the other groups.

In the first assessment (T1) we also tested users’ expectations toward online training and, in the second assessment (T2), the usability of the intervention (online training). Measures of expectations showed that the most preferred components were exercises enhancing the self-efficacy (70.5%) and relaxation techniques (66.3%). Therefore, in the e-stressless online training we focused on modules related to self-efficacy and relaxation. After the intervention, in T2, participants with high activity in the course rated its usability. The usability of the intervention in coping with stress was assessed as high (*M* = 3.84, *SD* = 1.01).

## Discussion

In the presented longitudinal study, the main aim was to test the efficiency of blended psychological intervention in employees’ stress reduction, more specifically the stress related to digital transformation. Because of reported high dropout rate of self-guided internet interventions (Hoerger, 2010; Rogala et al., 2016; Smoktunowicz et al., 2021) we decided to use the blended intervention, and combine self-guided online training addressing digital transformation stress with online interactive workshops with participants. The interactive workshops might have had additional social support function, which could increase self-efficacy (Hogan et al., 2002). We assumed that because the increase of self-efficacy raises a person’s ability to solve difficult tasks and endeavors and succeed in them for a long time (Gam et al., 2016), it consequently results in improvement in the ability to cope with stress (Cieslak et al., 2016; Gam et al., 2016).

To verify the effects of the intervention on digital transformation stress and more general work outcomes, namely general stress, self-efficacy at work, and burnout, we assessed these measures before (T1) and after (T2) the intervention. We compared five groups of participants depending on their participation in the workshop, willingness to participate, baseline level of stress and activity during the intervention (Zwarenstein et al., 2008; Patsopoulos, 2011; Loudon et al., 2015). The results indicated that in the group of participants who were active in the intervention the levels of digital transformation stress, negative emotions toward digital transformation and disengagement were lower after the intervention in comparison to the baseline level. These results, in our opinion, offered a preliminary confirmation of the positive effect of the blended intervention in reducing digital transformation stress. By lowering the level of burnout dimension—exhaustion—these results are also in line with our assumptions that this kind of psychological intervention may influence not only specific stress related to digital transformation but also more general work outcomes. The latter results are of great practical importance because disengagement is associated with the intention to resign from work and may have a tremendous effect on the available workforce (Bakker et al., 2005; Atanasoff and Venable, 2017; Willard-Grace et al., n.d.).

Although we assumed that the intervention should strengthen employees’ resources, namely self-efficacy, we did not observe significant increase in this variable. We believe that such changes may appear in some time distance and therefore the third measurement point would be necessary to evaluate such a prolonged change. Furthermore, it can be hypothesized that this type of intervention influences digital transformation stress rather by providing social support (Hogan et al., 2002; Cieślak et al., 2018) or by helping to deal with negative emotions (Hülsheger et al., 2013; Ninaus et al., 2015), than by changing self-efficacy. These alternative mechanisms should be verified in further studies.

Our results also offer very important contribution for practice. Our intervention seems to be “fighting fire with fire,” because it significantly reduced the digital transformation stress by using online intervention [i.e., digital (ICT) solution]. Moreover, we successfully tested the concept of internet intervention using an open-source e-learning platform such as Moodle, which enabled users to self-develop an effective open access intervention without sophisticated IT knowledge. This platform offered also quite good user experience (UX) qualities and were positively evaluated by the participants representing a wide range of business sectors; therefore the sample of employees was very heterogeneous. In comparison to other online interventions (Cieslak et al., 2016; Rogala et al., 2016; Smoktunowicz et al., 2019), our blended intervention had similar results in effectiveness within the active group of participants, with a lower dropout rate (18%) vs. (c.a. 80%) in other online interventions (Rogala et al., 2016; Smoktunowicz et al., 2019, 2021). Thus, the effect of blended intervention seems to have the potential to be available for both practitioners and wide range of users.

Additionally, in our study, in the follow-up assessment (T2) we measured the whole group of respondents, not only participants of the blended intervention. This approach allowed us to test the DTS score in ineligible groups. According to this approach, we can cautiously conclude that the lack of blended intervention has increased the level of digital transformation stress in comparison to active participation in interventions, whose DTS significantly decreased.

## Limitations

The present study has several limitations that need to be emphasized. Firstly, because of the COVID-19 pandemic, and the resulting online activities overload, participants may hesitate to engage in the additional Internet initiative, like online meetings, workshops and trainings. As a consequence, this factor could be one of the reasons for the high dropout rate and the low activity of the 4th group. Although the differences between active and control groups are not significant, we consider using a randomized control trial (RCT) approach in the future studies (Rogala et al., 2016; Smoktunowicz et al., 2019, 2021).

Secondly, a related limitation was finding balance in using digital solutions, namely the online intervention, as a digital transformation stress countermeasure, especially in the situation where people spend a lot of time in front of the computer out of necessity. Although participation in the intervention might be demanding due to the lockdown difficulties and tiredness while working online for the whole day before, the activity during workshops was successful. Monitoring of online training frequency of the participants revealed that they completed the majority of proposed exercises. However, using this intervention in a group of employees working in a traditional way might be a good control group in the future research.

The next limitation was lack of a possibility to receive objective measures of the level of digital transformation in a participants’ organization and necessity to rely on self-report measures. Possibly, some participants might have overestimated the level of digital transformation in their organization. For future research, we should purposefully select the organizations to invite participants to the study.

Finally, we were not able to observe the prolonged effects of the interventions that would enable us to evaluate the stability of observed effects in the long term, with 3 measurements of dependent variables (stress in the workplace, digital transformation stress, digital transformation attitudes). Finally, in the further research we should consider examining intervention effect in different cultural contexts, for generalization of results. Although recent meta-analysis on effectiveness of Internet -based CBT interventions confirmed its effectiveness in different cultural context (Andersson et al., 2019) it is important to better understand factors that may limit its usability.

## Conclusion

This study offers both theoretical and practical contributions. It confirmed the usefulness of ICT demands and employees’ resources model in the context of the digital transformation stress and digital transformation attitude. The blended intervention with e-stressless online training is an effective program enhancing the well-being of professionals affected by ICT demands increasing during the accelerated digital transformation in the workplace. Being broadly accessible to employees who currently work under DT demands, the proposed, blended intervention offers substantial psychological and social support, especially in the situation of remote work.

## Data Availability Statement

The raw data supporting the conclusions of this article will be made available by the authors, without undue reservation.

## Ethics Statement

The research protocol was approved by the Ethical Committee of the SWPS University of Social Sciences and Humanities, Warsaw, Poland, number of decisions: 47/2020, 50/2020, 3/2021, 8/2021. The study was conducted in compliance with ethical standards adopted by the American Psychological Association (APA, 2010). Accordingly, prior to participation, all participants were informed about the general aim of the research and the anonymity of their data. The patients/participants provided their written informed consent to participate in this study. Participation was voluntary, and the participants did not receive compensation for their participation in the study.

## Author Contributions

EM-T: research concept, project preparation, organization, planning, scales development methodology, writing the original draft, intervention content development, and design of figures. SB and EM-T: data curation, formal analysis, interpretation of results, writing the manuscript, editing, and revision. EM-T and KS: project administration and first abstract writing. SB and KS: scale English back translation. EM-T and JP: intervention diagram preparation. All authors contributed to the article and approved the submitted version.

## Conflict of Interest

The authors declare that the research was conducted in the absence of any commercial or financial relationships that could be construed as a potential conflict of interest.

## Publisher’s Note

All claims expressed in this article are solely those of the authors and do not necessarily represent those of their affiliated organizations, or those of the publisher, the editors and the reviewers. Any product that may be evaluated in this article, or claim that may be made by its manufacturer, is not guaranteed or endorsed by the publisher.
