# Supplementary material for: Blended Online Intervention to Reduce Digital Transformation Stress by Enhancing Employees’ Resources in COVID-19
Source: Front Psychol. 2022 Mar 22;13:732301. doi: 10.3389/fpsyg.2022.732301 (PMC8982670; doi:10.3389/fpsyg.2022.732301)
Supplement: Supplementary file 1 [file Data_Sheet_1.PDF]

Supplementary material

The following tables include descriptive statistics (means, and standard errors) and pairwise comparisons between intervention groups for significant main effect of intervention groups for all dependent variables in the study. Additionally, for significant interactions revealed for Digital Transformation Stress, Digital Transformation Attitude Scale – Negative Affect and Burnout – Exhaustion, we present statistics and pairwise comparisons between intervention groups separately for pre- and post- intervention measurement.

Table 1. *Pairwise comparisons of average level of digital transformation stress between intervention groups*

Main effect of intervention group on digital transformation stress - pairwise comparisons between interventions groups

Intervention group	Mean	SE	Significant differences (*p* < .05)
Group 1: Not assigned not willing with low DTS	1.889	.051	G1 vs G2, G1 vs G3, G1 vs G4, G1 vs G5
Group 2: Not assigned not willing high DTS	2.825	.057	G2 vs G1, G2 vs G3, G2 vs G4, G2 vs G5
Group 3: Not assigned but willing with low DTS	2.083	.045	G3 vs G1, G3 vs G2, G3 vs G4, G3 vs G5
Group 4: Assigned to intervention - passive	3.023	.060	G4 vs G1, G4 vs G2, G4 vs G3
Group 5: Assigned to intervention - active	3.110	.095	G5 vs G1, G5 vs G2, G5 vs G3

Table 2. *Pairwise comparisons of average level of digital transformation stress between intervention groups and measurement point (pre- vs post-intervention)*

Interaction of intervention group and measurement time on the level of digital transformation stress – comparison between intervention group separately in time points

Time	Intervention group	Mean	SE	Significant differences (*p* < .05)
Time 1	Group 1: Not assigned not willing with low DTS	1.757	.050	G1 vs G2, G1 vs G3, G1 vs G4, G1 vs G5
	Group 2: Not assigned not willing high DTS	2.947	.056	G2 vs G1, G2 vs G3, G2 vs G5
	Group 3: Not assigned but willing with low DTS	1.953	.044	G3 vs G1, G3 vs G2, G3 vs G4, G3 vs G5
	Group 4: Assigned to intervention - passive	3.064	.060	G4 vs G1, G4 vs G3
	Group 5: Assigned to intervention - active	3.226	.093	G5 vs G1, G5 vs G2, G5 vs G3
Time 2	Group 1: Not assigned not willing with low DTS	2.022	.068	G1 vs G2, G1 vs G3, G1 vs G4, G1 vs G5
	Group 2: Not assigned not willing high DTS	2.702	.077	G2 vs G1, G2 vs G3, G2 vs G4, G2 vs G5

	Group 3: Not assigned but willing with low DTS	2.214	.060	G3 vs G1, G3 vs G2, G3 vs G4, G3 vs G5
	Group 4: Assigned to intervention - passive	2.982	.081	G4 vs G1, G4 vs G2, G4 vs G3
	Group 5: Assigned to intervention - active	2.995	.127	G5 vs G1, G5 vs G2, G5 vs G3

Table 3. *Pairwise comparisons of average level of Digital Transformation Attitude Scale – Proactive Behavior between intervention groups*

Main effect of intervention group on Digital Transformation Attitude Scale – Proactive Behavior - pairwise comparisons between interventions groups

Intervention group	Mean	SE	Significant differences ($p < .05$)
Group 1: Not assigned not willing with low DTS	2.692	.078	G1 vs G3, G1 vs G4, G1 vs G5
Group 2: Not assigned not willing high DTS	2.835	.087	G2 vs G3, G2 vs G4
Group 3: Not assigned but willing with low DTS	3.374	.069	G3 vs G1, G3 vs G2
Group 4: Assigned to intervention - passive	3.197	.093	G4 vs G1, G4 vs G2
Group 5: Assigned to intervention - active	3.065	.145	G5 vs G1

Table 4. *Pairwise comparisons of average level of Digital Transformation Attitude Scale – Positive Cognition between intervention groups*

Main effect of intervention group on Digital Transformation Attitude Scale – Positive Cognition - pairwise comparisons between interventions groups

Intervention group	Mean	SE	Significant differences ($p < .05$)
Group 1: Not assigned not willing with low DTS	3.919	.070	G1 vs G2, G1 vs G3
Group 2: Not assigned not willing high DTS	3.555	.078	G2 vs G1, G2 vs G3, G2 vs G4
Group 3: Not assigned but willing with low DTS	4.109	.061	G3 vs G1, G3 vs G2, G3 vs G4, G3 vs G5
Group 4: Assigned to intervention - passive	3.849	.083	G4 vs G2, G4 vs G3
Group 5: Assigned to intervention - active	3.774	.130	G5 vs G3

Table 5. *Pairwise comparisons of average level of Digital Transformation Attitude Scale – Negative Cognition between intervention groups*

Main effect of intervention group on Digital Transformation Attitude Scale – Negative Cognition - pairwise comparisons between interventions groups

Intervention group	Mean	SE	Significant differences (p < .05)
Group 1: Not assigned not willing with low DTS	2.879	.068	G1 vs G2, G1 vs G4, G1 vs G5
Group 2: Not assigned not willing high DTS	3.314	.076	G2 vs G1, G2 vs G3
Group 3: Not assigned but willing with low DTS	2.792	.059	G3 vs G2, G3 vs G4, G3 vs G5
Group 4: Assigned to intervention - passive	3.213	.080	G4 vs G1, G4 vs G3
Group 5: Assigned to intervention - active	3.499	.125	G5 vs G1, G5 vs G3

Table 6. *Pairwise comparisons of average level of Digital Transformation Attitude Scale – Negative Affect between intervention groups*

Main effect of intervention group on Digital Transformation Attitude Scale – Negative Affect - pairwise comparisons between interventions groups

Intervention group	Mean	SE	Significant differences (p < .05)
Group 1: Not assigned not willing with low DTS	2.252	.064	G1 vs G2, G1 vs G4, G1 vs G5
Group 2: Not assigned not willing high DTS	2.708	.072	G2 vs G1, G2 vs G3, G2 vs G4, G2 vs G5
Group 3: Not assigned but willing with low DTS	2.357	.056	G3 vs G2, G3 vs G4, G3 vs G5
Group 4: Assigned to intervention - passive	2.930	.076	G4 vs G1, G4 vs G3
Group 5: Assigned to intervention - active	2.997	.119	G5 vs G1, G5 vs G2, G5 vs G3

Table 7. *Pairwise comparisons of average level of Digital Transformation Attitude Scale – Negative Affect between intervention groups and measurement point (pre- vs post-intervention)*

Interaction of intervention group and measurment time on the level of Digital Transformation Attitude Scale – Negative Affect – comparison between intervention group separately in time points -

Time	Intervention group	Mean	SE	Significant differences (p < .05)

Time 1	Group 1: Not assigned not willing with low DTS	2.237	.075	G1 vs G2, G1 vs G4, G1 vs G5
	Group 2: Not assigned not willing high DTS	2.667	.084	G2 vs G1, G2 vs G3, G2 vs G4, G2 vs G5
	Group 3: Not assigned but willing with low DTS	2.338	.066	G3 vs G2, G3 vs G4, G3 vs G5
	Group 4: Assigned to intervention - passive	2.925	.089	G4 vs G1, G4 vs G2, G4 vs G3
	Group 5: Assigned to intervention - active	3.199	.140	G5 vs G1, G5 vs G2, G5 vs G3
Time 2	Group 1: Not assigned not willing with low DTS	2.268	.076	G1 vs G2, G1 vs G4, G1 vs G5
	Group 2: Not assigned not willing high DTS	2.749	.085	G2 vs G1, G2 vs G3,
	Group 3: Not assigned but willing with low DTS	2.377	.067	G3 vs G2, G3 vs G4, G3 vs G5
	Group 4: Assigned to intervention - passive	2.934	.090	G4 vs G1, G4 vs G3
	Group 5: Assigned to intervention - active	2.796	.141	G5 vs G1, G5 vs G3

Table 8. Pairwise comparisons of average level of Self-Efficacy between intervention groups

Main effect of intervention group on Self-Efficacy - pairwise comparisons between interventions groups

Intervention group	Mean	SE	Significant differences ($p < .05$)
Group 1: Not assigned not willing with low DTS	3.914	.055	G1 vs G2, G1 vs G4, G1 vs G5
Group 2: Not assigned not willing high DTS	3.468	.062	G2 vs G1, G2 vs G3
Group 3: Not assigned but willing with low DTS	3.940	.049	G3 vs G2, G3 vs G4, G3 vs G5
Group 4: Assigned to intervention - passive	3.550	.066	G4 vs G1, G4 vs G3
Group 5: Assigned to intervention - active	3.630	.103	G5 vs G1, G5 vs G3

Table 9. Pairwise comparisons of average level of Burnout – Disengagement between intervention groups

Main effect of intervention group on Burnout – Disengagement - pairwise comparisons between interventions groups

Intervention group	Mean	SE	Significant differences ($p < .05$)
Group 1: Not assigned not willing with low DTS	2.131	.050	G1 vs G2, G1 vs G5
Group 2: Not assigned not willing high DTS	2.331	.056	G2 vs G1, G2 vs G3
Group 3: Not assigned but willing with low DTS	2.051	.044	G3 vs G2, G3 vs G4, G3 vs G5
Group 4: Assigned to intervention - passive	2.259	.060	G4 vs G3, G4 vs G5
Group 5: Assigned to intervention - active	2.506	.097	G5 vs G1, G5 vs G3, G5 vs G4

Table 7. Pairwise comparisons of average level of Burnout – Disengagement between intervention groups and measurement point (pre- vs post-intervention)

Interaction of intervention group and time point on the level of Burnout – Disengagement – comparison between intervention group separately in time points

Time:	Intervention group	Mean	SE	Significant differences ($p < .05$)
Time 1	Group 1: Not assigned not willing with low DTS	2.125	.055	G1 vs G2, G1 vs G5
	Group 2: Not assigned not willing high DTS	2.353	.062	G2 vs G1, G2 vs G3, G2 vs G5
	Group 3: Not assigned but willing with low DTS	1.998	.048	G3 vs G2, G3 vs G4, G3 vs G5
	Group 4: Assigned to intervention - passive	2.219	.065	G4 vs G3, G4 vs G5
	Group 5: Assigned to intervention - active	2.621	.106	G5 vs G1, G5 vs G2, G5 vs G3, G5 vs G4
Time 2	Group 1: Not assigned not willing with low DTS	2.137	.056	G1 vs G2, G1 vs G5
	Group 2: Not assigned not willing high DTS	2.310	.062	G2 vs G1, G2 vs G3
	Group 3: Not assigned but willing with low DTS	2.104	.049	G3 vs G2, G3 vs G4, G3 vs G5
	Group 4: Assigned to intervention - passive	2.298	.066	G4 vs G3
	Group 5: Assigned to intervention - active	2.391	.107	G5 vs G1, G5 vs G3

Table 9. Pairwise comparisons of average level of Burnout – Exhaustion between intervention groups

Main effect of intervention group on Burnout – Disengagement - pairwise comparisons between interventions groups

Intervention group	Mean	SE	Significant differences ($p < .05$)
Group 1: Not assigned not willing with low DTS	2.184	.050	G1 vs G2, G1 vs G4, G1 vs G5
Group 2: Not assigned not willing high DTS	2.469	.056	G2 vs G1, G2 vs G3
Group 3: Not assigned but willing with low DTS	2.222	.044	G3 vs G2, G3 vs G4, G3 vs G5
Group 4: Assigned to intervention - passive	2.509	.059	G4 vs G1, G4 vs G3
Group 5: Assigned to intervention - active	2.678	.095	G5 vs G1, G5 vs G3
